# Spring temperature drives phenotypic selection on plasticity of flowering time

**DOI:** 10.1098/rspb.2023.0670

**Published:** 2023-09-06

**Authors:** Alicia Valdés, Pieter A. Arnold, Johan Ehrlén

**Affiliations:** ^1^ Department of Ecology, Environment and Plant Sciences, Stockholm University, 106 91 Stockholm, Sweden; ^2^ Bolin Centre for Climate Research, Stockholm University, Stockholm, Sweden; ^3^ Division of Ecology and Evolution, Research School of Biology, The Australian National University, Canberra, Australian Capital Territory 2600, Australia

**Keywords:** climate change, climate sensitivity, evolutionary responses, fitness, plant phenology, reaction norms

## Abstract

In seasonal environments, a high responsiveness of development to increasing temperatures in spring can infer benefits in terms of a longer growing season, but also costs in terms of an increased risk of facing unfavourable weather conditions. Still, we know little about how climatic conditions influence the optimal plastic response. Using 22 years of field observations for the perennial forest herb *Lathyrus vernus*, we assessed phenotypic selection on among-individual variation in reaction norms of flowering time to spring temperature, and examined if among-year variation in selection on plasticity was associated with spring temperature conditions. We found significant among-individual variation in mean flowering time and flowering time plasticity, and that plants that flowered earlier also had a more plastic flowering time. Selection favoured individuals with an earlier mean flowering time and a lower thermal plasticity of flowering time. Less plastic individuals were more strongly favoured in colder springs, indicating that spring temperature influenced optimal flowering time plasticity. Our results show how selection on plasticity can be linked to climatic conditions, and illustrate how we can understand and predict evolutionary responses of organisms to changing environmental conditions.

## 1. Introduction

Individual organisms can adapt to varying environmental conditions through phenotypic plasticity, which allows a rapid adjustment to short-term variation in environmental conditions [1,2]. Plasticity of a particular trait might vary among individuals and this variation can be genetically based [3]. The optimal pattern of plasticity of a trait, and thus natural selection acting on plasticity, depends on the distribution of environmental conditions, and on the relationship between current and future conditions [4–7]. Organisms are therefore likely to adapt to altered environmental conditions by genetic changes in their plastic responses. Assessing how plasticity varies among individuals, and identifying the environmental conditions that make plastic responses adaptive, non-adaptive or maladaptive, is thus fundamental to understand the evolution of plasticity and to predict how organisms respond to changes in the environment [8,9].

In seasonal environments where growing seasons are on average short and vary in length among years, phenotypic plasticity in development rates enables organisms to match key life-history events to suitable conditions. In these environments, the optimal response of development to increasing temperature in early spring is likely to be the result of a trade-off. A high plasticity leads to an earlier development, which is associated with benefits in terms of increased opportunities to capitalize on favourable conditions, but also with costs, as highly plastic individuals in some years might express a suboptimal phenotype that faces unfavourable climatic conditions, in terms of low temperatures and spring frosts [10–12]. Because the relationship between early spring temperature conditions and later conditions that are important for fitness might differ among years, we expect the optimal plastic response to vary among years. We thus expect natural selection to act not only on the actual timing of reproduction expressed by individuals in each year [13,14], but also on plasticity of development. For example, in years with cold springs natural selection might favour low plasticity, because an early development would increase the risk of facing unfavourable conditions, while in years with warm springs high plasticity might be favoured because an early development allows individuals to capitalize on favourable conditions for growth and flowering during a longer period.

Plastic responses of traits that are expressed multiple times during the lifetime of an individual can be described by individual reaction norms, which are the relationships between the environmental conditions experienced by individuals during their lifetime and the phenotypic values expressed under these conditions [3,15–17]. For example, the change in timing of reproduction with changes in spring temperature can be assessed by estimating the intercept and slope of the relationship between time of reproduction and yearly spring temperature. The central intercept and the slope of the reaction norm represent, respectively, the average timing and plasticity of timing of reproduction. A higher intercept indicates that an individual is reproducing on average later, and a steeper slope indicates that timing of reproduction differs more with a given temperature difference (i.e. advances more days per degree of temperature increase). Reaction norm intercepts and slopes might differ among individuals, and can be regarded as quantitative traits that are subject to selection [18]. Given that phenotypic mean trait values and magnitudes of trait plasticity might often be correlated [12], it is important to separate direct selection on plasticity (i.e. on the slope of the reaction norm) from selection acting indirectly through association with mean trait value [19]. Among-individual variation in plasticity in timing of reproduction in natural populations has been demonstrated in both animals [20–24] and plants [25–27]. Studies examining phenotypic selection on phenological plasticity in response to temperature have found selection for increased [20,22,26], intermediate levels [28], as well as decreased plasticity [25,27], the latter suggesting that current levels of plasticity might be maladaptive. A few studies have also examined how environmental factors, such as soil chemistry, food availability and experimental heating, influence selection on plasticity of the timing of reproduction [20,26,29,30]. However, we still know very little about how selection on plasticity of timing of reproduction in natural populations is linked to climatic conditions.

In this study, we quantified phenotypic selection on individual reaction norms of flowering time to temperature in the spring-flowering perennial herb *Lathyrus vernus*, and investigated how among-year variation in selection was related to spring temperatures. We used 22 years of field observations of first flowering day, above-ground size and the number of intact seeds produced for 837 permanently marked individuals. Previous studies with this species have shown that temperature and, to a lesser extent, precipitation in spring influence first flowering day, and that also selection on flowering time is influenced by climatic conditions during spring [13]. Here, we focus on thermal reaction norms of first flowering day and test three hypotheses:

(1) Both the mean response (central intercept) and the slope of reaction norms of flowering time to spring temperature vary among individuals. To examine this, we compared three models of the relationship between first flowering day and April temperature, assuming that individuals do not differ, differ in only intercept, or differ in both intercept and slope of their reaction norms, respectively.
(2) There is phenotypic selection on both the intercept and the slope of reaction norms, i.e. on both average flowering time and on flowering time plasticity. To examine this, we estimated the covariance of mean individual fitness (mean number of intact seeds) with intercept and slope of individual reaction norms, and assessed total and direct selection.
(3) Optimal flowering time plasticity, and thus the outcome of yearly events of selection, depend on temperature conditions during spring. Specifically, we predicted that more plastic individuals have higher fitness in warmer springs, while less plastic individuals have higher fitness in colder springs with less favourable conditions for growth and flowering. To test this, we examined if fitness for each individual flowering event was influenced by the interaction between individual flowering time plasticity and spring temperature.

## 2. Methods

### (a) Study system

*Lathyrus vernus* is a long-lived understory herb with an average conditional life span of flowering individuals estimated to 44.3 years [31]. This species lacks means of vegetative propagation and different individuals in the population thus constitute different genotypes. The first flowering event usually occurs after 10–15 years and after reaching a certain size (above-ground volume ≥ 230 mm^3^), although plants above this size threshold frequently skip flowering in some years [13]. The shoots of the next season are initiated and flower buds differentiated during the summer before flowering [32]. In March–April, one to several shoots grow out from the over-wintering shoot buds on the rhizome, and flowering starts about one month later [33,34]. The large pink-purple flowers are pollinated by bumblebees (*Bombus* spp.). *L. vernus* is self-compatible, but it lacks mechanisms for autogamy (J. Ehrlén, unpublished data). Individual flowering time influences the intensity of interactions with animals that may act as selective agents [35]. Early-flowering plants are more damaged by roe deer (*Capreolus capreolus*) than late-flowering individuals, but still produce on average more seeds per flower, possibly due to higher light and pollen availability early during spring [34,36]. Plants produce a small number (mean ± s.d. = 5.0 ± 1.8 seeds per fruit) of large seeds (mean ± s.d. = 12.0 ± 3.5 mg), and recruitment from seeds is common [37,38]. The pre-dispersal seed predator beetle *Bruchus atomarius* often damages developing seeds.

### (b) Data collection

The study was carried out 1987–1996 and 2006–2017 in a *L. vernus* population located in a deciduous forest in Tullgarn, southeast Sweden (58.9496 N, 17.6097 E) [13]. All flowering individuals in an 825 m^2^ plot were permanently marked in 1987, and surveyed each year to 1996. No recordings were made 1997–2005. In 2006, a new, non-overlapping set of individuals in a different plot of 162 m^2^ within the same population were marked, and surveyed to 2017. In each season, recordings started when shoots initiated growth in April, and were performed every fifth day until all plants had finished flowering. The number of buds, open flowers, scars from aborted flowers and fruits were recorded at each visit. We also recorded if shoots had been grazed. Every year, new flowering individuals appearing in the plots were marked and included in the study. In total we followed 837 individuals, corresponding to different genotypes (607 from 1987 to 1996, and 230 from 2006 to 2017), and recorded 2478 flowering events (mean number of events per individual ± s.d. = 3.3 ± 2.6).

We used first flowering day, i.e. the day when the first flower was fully unfolded with the banner petal folded upwards, as a measure of individual flowering phenology. Using information about the presence of open flowers from each visit, we first determined the 5-day interval during which an individual had started flowering. We then estimated the most likely first flowering day from this interval, using information about the size of the most developed bud at the beginning of the interval, and the number of open flowers at the end of the interval [13]. To account for leap years, first flowering day was converted from calendar dates to number of days after the vernal equinox. Excluding individuals subjected to grazing (the invisible fraction [39]) from the analyses is likely to result in inaccurate estimates of selection [40]. In plants that were grazed before we observed that the first flower had opened, we therefore estimated first flowering day based on bud sizes in previous recordings if 50% or more of the shoots were grazed [13]. These plants might either have flowered and were grazed before we observed the opening of the first flower, or were grazed prior to opening of the first flower. In the latter case, we calculated an ‘expected’ first flowering day to account for that grazers are potentially important agents of selection on flowering time [35].

At the time of seed ripening, we measured vegetative size, recorded whether individuals had produced any seeds, and counted the numbers of mature fruits, intact seeds and seeds damaged by seed predator beetles in seed-producing individuals. Vegetative size was estimated as the above-ground volume of each plant in mm^3^, calculated as sum of the volumes of all shoots (volume = π × shoot radius^2^ × shoot height [13]). In fruits that had not yet opened, intact and damaged seeds were counted in the field (seeds with larval entrance holes were considered damaged). In fruits that had opened prior to the recording, we estimated the numbers of intact and damaged seeds based on the number of placentas and larval entrance holes [13]. We used the number of intact seeds produced by an individual in one year as a measure of fitness. We calculated two different measures of mean fitness across years for each individual: mean fitness per flowering event (the number of intact seeds produced over all years divided by the number of years in which the plant was flowering), and mean fitness per year of study (the number of intact seeds produced over all years divided by the number of years that the plant was included in the study). These two measures were highly correlated (Pearson's *r* = 0.87).

We used the average daily mean temperature of April as a measure of spring temperature [13]. This value was calculated as the average of temperatures from the two nearest meteorological stations (Swedish Meteorological and Hydrological Institute, www.smhi.se). As our study focused on among-year variation in temperatures and did not include substantial spatial variation, we considered that the use of weather-station data was adequate and that there was no need to use down-scaled climatic data. We used April temperature because it represents the period when most individuals initiate growth, and it determines the timing of bud development and flowering start. In April, *L. vernus* growth is mainly limited by temperature, as the seasonal snow cover is gone, there is no shortage of water and light conditions are favourable. During the study period, mean temperature was also strongly negatively correlated with the number of days with freezing temperatures during April (*r* = −0.79). Mean temperature during spring has previously been shown to be the most important climatic predictor for among-year variation in first flowering day [13]. Temperature stress or drought are not likely to be important in our study as temperatures during spring and early summer are mild and availability of water high.

### (c) Statistical analyses

We used all 837 individuals that flowered during any of the study years for our analyses, including also those for which first flowering day was available for only one or a few years. The choice to include all individuals was based on that a previous simulation study found that this approach increased the power to detect individual variation in linear reaction norms and reduced the confidence intervals around variance component estimates [41]. The suggested reason for this is that, albeit individuals with only one observation provide no direct information about the slope of the reaction norm, slopes and intercepts are estimated simultaneously in random regression models, and individuals with only one observation provide information with respect to some model parameters to be estimated. Still, individuals with only one or a few records might differ from individuals with more observations, and we therefore performed also additional analyses including individuals flowering in at least 2, 3 or 4 years to examine how estimates were influenced by different types of censoring. Descriptive statistics for all variables used in the analyses are shown in electronic supplementary material, table S1, and a histogram of the number of flowering events per individual is included as electronic supplementary material, figure S1. Values of April temperature were mean-centred prior to analyses, meaning that reaction norm intercepts reflect average trait values [42,43]. We chose to focus the analyses on thermal reaction norms because temperature had a much stronger effect on first flowering day than precipitation [13], and because we wanted to avoid overly complex models.

In order to examine the existence of among-individual variation in thermal reaction norms of flowering time (Hypothesis 1), we fitted three increasingly complex linear mixed models with Gaussian distributions and year as a random intercept (to account for multiple recordings of first flowering day at each temperature). The first model assumed no among-individual variation around the average population-level reaction norm, and included first flowering day as the response variable and April temperature as a fixed effect. The second model assumed among-individual variation in reaction norm intercept, and included also random intercepts for individuals. The third model assumed among-individual variation in reaction norm intercept and slope, and included both random intercepts and random slopes for individuals (random regression mixed model, RRMM [42–44]). We then investigated the support for among-individual variation in reaction norms by comparing the performances of the three models, using the Bayesian leave-one-out information criterion (LOOIC) [45]. If the third model (RRMM) has the highest performance (lowest LOOIC), we interpret this as evidence of among-individual variation in both intercept and slope of reaction norms. We also repeated these analyses using either variance in April temperature or the number of days below zero in April instead of mean April temperature, in order to examine the existence of among-individual variation in reaction norms of first flowering day to variance in spring temperature and to the frequency of very cold days, respectively.

From the output of the RRMM, we estimated the posterior distribution of best linear unbiased predictors (BLUPs) for each individual, and then used the average BLUPs over all samples of the model as estimates of each individual's reaction norm slope and intercept. The BLUP intercept and slope estimates describe the difference in individual intercept and slope relative to the population average [43].

In order to examine if among-individual variation in thermal reaction norms of flowering time is under selection (Hypothesis 2), we estimated phenotypic selection on individual reaction norm intercept and slope using bivariate RRMMs with first flowering day and mean fitness across years each as response variables [19]. We fitted these models for mean fitness both per flowering event and per year of study separately. Selection on plasticity based on the first measure (per flowering event) would only account for differences in reproductive success during reproductive events, while selection on plasticity based on the second measure (per year of study) also accounts for differences in the frequency of flowering events. First flowering day was modelled with a Gaussian distribution and mean fitness was modelled with a negative binomial distribution. The models included a fixed effect of April temperature on first flowering day, a fixed effect of plant size (mean-centred, square-root-transformed above-ground volume) on fitness, a random intercept of year on first flowering day, and random intercepts and random slopes for individuals. Plant size was included to account for the fact that larger plants tend to have an earlier first flowering day, and models therefore examine among-individual variation in thermal reaction norms of flowering time while accounting for differences in plant size. These models allow assessment of both direct and indirect selection from estimates of the covariance of fitness with reaction norm intercepts and slopes, based on the classic Lande–Arnold selection framework [46], that have been adapted for determining selection on plasticity (see additional details in §4 of [19] and the paragraph below). Models without plant size gave similar results (not shown).

From each of the two bivariate RRMMs (based on either mean fitness per flowering event or per year of study), we calculated selection differentials and gradients for reaction norm intercepts and slopes. Selection differentials represent the total selection on reaction norm intercepts and slopes (including both direct and indirect selection), and were calculated as the covariances between fitness, and intercepts and slopes. Selection gradients represent the direct selection on intercept and slope respectively, and were calculated by correcting for the intercept–slope covariance using the product of the selection differentials and the inverse variance–covariance matrix of intercepts and slopes [19,46]. We calculated posterior modes and highest posterior density (HPD) intervals for both selection differentials and gradients. As the reaction norm slopes of flowering time to spring temperature are negative (i.e. at higher temperatures, flowering starts earlier and first flowering day values are lower), negative and positive selection gradients on reaction norm slopes indicate selection for increased (steeper slope) and decreased plasticity (shallower slope), respectively [19].

To test if differences in yearly events of selection on thermal plasticity of flowering time are linked to among-year variation in spring temperatures (Hypothesis 3), we fitted a generalized linear mixed model (zero-inflated negative binomial distribution) with fitness for each individual and year as the response variable, and April temperature, the BLUP slope estimate (as a measure of plasticity) and their interaction as fixed effects. We also included a fixed effect of plant size on fitness to account for plant condition. Individual was included as a random intercept, to account for multiple recordings of fitness for each individual. Using average BLUPs in statistical analyses, although previously criticized [47,48], has been shown to produce less biased estimates than analyses carrying forward uncertainty in BLUP values [49]. In the model that we used, an effect of the interaction April temperature × BLUP slope estimate is interpreted as evidence of that differences in spring temperature are associated with differences in phenotypic selection on thermal plasticity of flowering time. Ideally, we would also include a fixed effect of the BLUP intercept estimate in this model to account for differences in individual reaction norm intercept, but due to the high correlation among intercepts and slopes (see Results), this would lead to multicollinearity and low statistical power. To circumvent this problem, we therefore regressed the BLUP slope estimate on the BLUP intercept estimate to obtain a measure of plasticity adjusted for differences in average flowering time (residuals). We then fitted a model analogous to the one described above but using residuals instead of BLUP slope estimates.

All statistical analyses were carried out in R v. 4.0.3 [50]. Models were fitted in a Bayesian setting using the R package brms [51] with default priors. For each model, we ran the Markov chain Monte Carlo (MCMC) with four chains for at least 4000 iterations (sometimes more iterations were needed due to warnings), with the first 1000 used as burn-in and a thinning rate of 2, giving at least 6000 MCMC samples for inference. We visually inspected the chains for convergence, confirmed that the scale-reduction factor (*R*_hat_) was lower than 1.05, and ensured that the minimum effective sample size (*N*_eff_/*N*) was larger than 0.1 [52]. We assessed model fit using graphical posterior predictive checks and Bayesian *R*^2^ [53]. Model evaluation results and graphical posterior predictive checks are shown in electronic supplementary material, figures S2–S16.

## 3. Results

**Figure 1.**
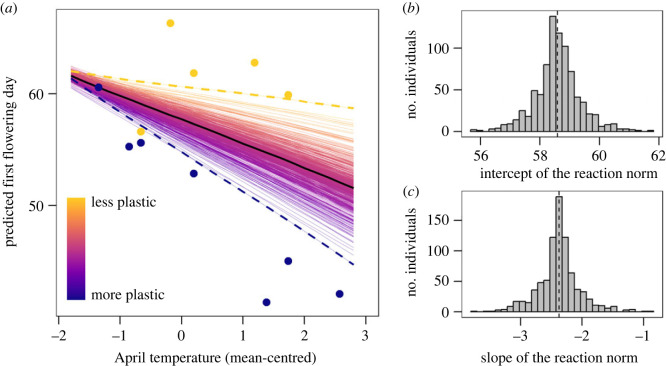
Among-individual variation in thermal plasticity of flowering time in *Lathyrus vernus*. (*a*) Predicted reaction norms of flowering time to spring temperature, based on a random regression mixed model of first flowering day on mean-centred April temperature, including both random intercepts and random slopes for individuals (table 1C). Thin lines represent the predicted reaction norms for each of 837 *L. vernus* individuals, coloured according to their thermal plasticity (i.e. to their reaction norm slope). The thick black line represents the average population-level reaction norm. For the least and most plastic individuals, reaction norms are highlighted with thicker, dashed lines, and raw data points of these individuals are shown. (*b*) Frequency of individual values of reaction norm intercept (BLUP intercept values + population average). (*c*) Frequency of individual values of reaction norm slope (BLUP slope values + population average). In (*b*,*c*), vertical dashed lines indicate population means.

First flowering day varied among years and among individuals within years (2478 flowering events; electronic supplementary material, table S1). Among-year variation was related to differences in mean April temperature (figure 1*a*). We found evidence of among-individual variation in both intercept and slope of reaction norms to mean April temperature. A model of first flowering day against April temperature including variation in both reaction norm intercept and slope among individuals was more supported than models assuming that individuals differed only in intercept, or did not differ at all (figure 1*a* and table 1). Reaction norm intercepts, representing average flowering time across years estimated from this model, ranged from 55.8 to 61.7 days after the vernal equinox (figure 1*b*), and reaction norm slopes, representing flowering time plasticity, ranged from −3.8 to −0.9 days/°C (figure 1*c*). There was a strong correlation between reaction norm intercepts and slopes, and individuals that on average flowered earlier were also more plastic (table 1C). We found evidence of among-individual variation in both intercept and slope of reaction norms to the frequency of days below zero in April, but only in the intercepts of reaction norms to variance in April temperature (electronic supplementary material, figure S17).

**Table 1.** Results of linear mixed models with Gaussian distributions testing for the existence of among-individual variation in flowering time plasticity. Year was included as a random intercept in all models (number of levels: 22). Models test the effect of mean-centred April temperature on first flowering day and include no random effects of individuals (A), random intercepts for individuals (B), and both random intercepts and random slopes for individuals (number of levels: 837) (C). Means and standard deviations (s.d.) of the posterior distribution as well as two sided 95% credible intervals (CI) are shown. Bayesian *R*^2^ and Bayesian leave-one-out information criterion (LOOIC) are shown for each model.

			(A) Bayesian *R*^2^ = 0.584				(B) Bayesian *R*^2^ = 0.632				(C) Bayesian *R*^2^ = 0.646			
			LOOIC = 14759.6				LOOIC = 14668.3				LOOIC = 14641.9			
			posterior mean	posterior s.d.	95% CI		posterior mean	posterior s.d.	95% CI		posterior mean	posterior s.d.	95% CI	
					lower	upper			lower	upper			lower	upper
group-level effects	individual	s.d. (intercept)	—	—	—	—	1.601	0.141	1.319	1.876	1.555	0.149	1.260	1.842
		s.d. (slope)	—	—	—	—	—	—	—	—	0.783	0.132	0.533	1.046
		correlation (intercept, slope)	—	—	—	—	—	—	—	—	0.800	0.135	0.492	0.990
	year	s.d. (intercept)	5.161	0.834	3.801	7.077	5.135	0.850	3.758	7.013	5.160	0.858	3.829	7.209
population-level effects		intercept	58.524	1.138	56.212	60.692	58.561	1.107	56.437	60.766	58.591	1.118	56.488	60.862
		April temperature	−2.455	0.830	−4.187	−0.851	−2.431	0.805	−4.004	−0.863	−2.375	0.831	−4.050	−0.737

**Figure 2.**
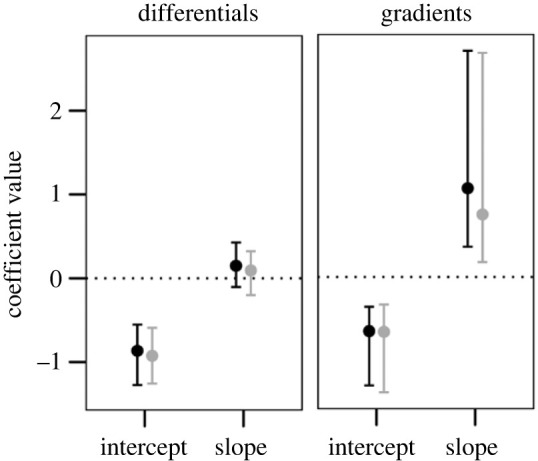
Selection differentials and gradients for intercepts and slopes of thermal reaction norms of flowering time in *Lathyrus vernus*, estimated using the models in table 2. Points indicate coefficient values (posterior modes), and error bars indicate highest posterior density (HPD) intervals (see electronic supplementary material, table S2, for values). Values for models using mean fitness per flowering event and per year of study are shown in black and grey, respectively.

There was direct selection for a lower responsiveness of flowering time to temperature, i.e. a positive selection gradient for reaction norm slopes, as well as direct selection for an earlier average flowering (figure 2 and electronic supplementary material, table S2). This was true in models based on mean fitness per flowering event as well as in models based on mean fitness per year of study (figure 2, table 2 and electronic supplementary material, table S2). Selection differentials indicated total selection for an earlier average flowering, but no total selection on plasticity (figure 2 and electronic supplementary material, table S2).

**Table 2.** Results of bivariate random regression mixed models with first flowering day (FFD) (Gaussian distribution) and mean fitness across years (negative binomial distribution) as response variable. Models were fitted for (A) mean fitness per flowering event and (B) mean fitness per year of study. Models include a fixed effect of mean-centred April temperature on first flowering day, a fixed effect of plant size (square-root transformed above-ground volume) on fitness, a random intercept of year (number of levels: 22) on first flowering day, and random intercepts and random slopes for individuals (number of levels: 837). Means and standard deviations (s.d.) of the posterior distribution as well as two sided 95% credible intervals (CI) are shown. Bayesian *R*^2^ values are shown for each response variable and model.

			(A) mean fitness per flowering event				(B) mean fitness per year of study			
			Bayesian *R*^2^ first flowering day = 0.649				Bayesian *R*^2^ first flowering day = 0.648			
			Bayesian *R*^2^ fitness = 0.939				Bayesian *R*^2^ fitness = 0.941			
			posterior mean	posterior s.d.	95% CI		posterior mean	posterior s.d.	95% CI	
					lower	upper			lower	upper
group-level effects	individual	s.d. (intercept of FFD)	1.665	0.148	1.377	1.964	1.651	0.148	1.365	1.936
		s.d. (slope of FFD)	0.804	0.133	0.546	1.068	0.801	0.132	0.541	1.062
		s.d. (intercept of fitness)	1.240	0.049	1.150	1.341	1.199	0.050	1.107	1.302
		correlation (intercept of FFD, slope of FFD)	0.641	0.135	0.352	0.873	0.688	0.131	0.407	0.905
		correlation (intercept of FFD, intercept of fitness)	−0.448	0.077	−0.594	−0.290	−0.466	0.075	−0.606	−0.311
		correlation (slope of FFD, intercept of fitness)	0.162	0.134	−0.103	0.427	0.053	0.138	−0.214	0.323
	year	s.d. (intercept of FFD)	5.219	0.882	3.821	7.152	5.223	0.860	3.840	7.158
population-level effects		intercept of FFD	58.644	1.124	56.467	60.869	58.630	1.106	56.428	60.835
		intercept of fitness	0.791	0.053	0.686	0.898	0.132	0.057	0.016	0.242
		April temperature on FFD	−2.396	0.831	−4.046	−0.794	−2.423	0.832	−4.054	−0.797
		vegetative size on fitness	0.040	0.004	0.032	0.047	0.054	0.004	0.046	0.063

**Figure 3.**
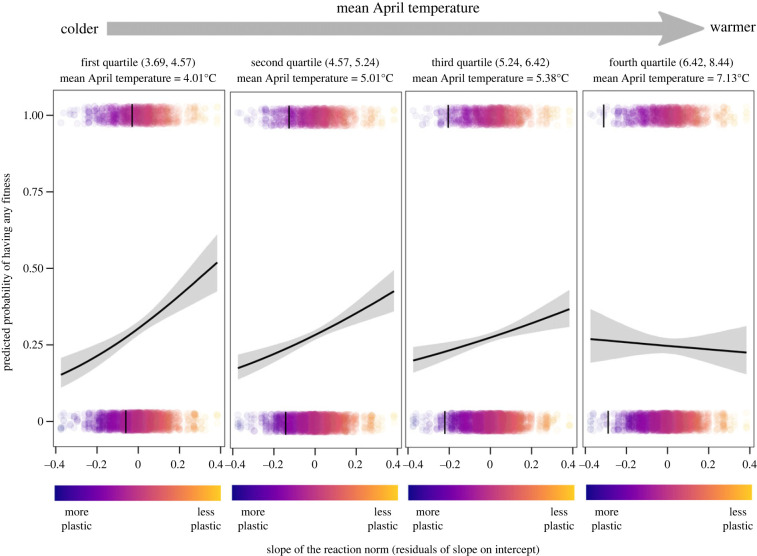
Effect of spring temperature on phenotypic selection on thermal plasticity of flowering time in *Lathyrus vernus*. Lines show the relationship between the measure of thermal plasticity, adjusted for differences in average flowering time (residuals of the BLUP slope estimate on the BLUP intercept estimate) and the predicted probability of having any fitness at different April temperatures. The four vertical panels show data for four quartiles of the distribution of April temperatures (interval values are indicated above each panel), going from colder to warmer temperatures (from left to right). Fitness was estimated by the number of intact seeds, but effects of temperature on the covariation between reaction norm slopes and fitness only acted via the probability of producing any seeds. Lines show predicted marginal effects (effects of the focal variables when holding the non-focal variables constant at their means) and confidence intervals from a generalized linear mixed model with zero-inflated negative binomial distribution, including also a fixed effect of plant size and a random intercept for individual, and represent the predicted effect of plasticity on the probability of having any intact seeds (1 − the predicted zero-inflation probability of fitness; see table 3 for the full model output) for the mean April temperature of each quarter (indicated above each panel). Points are raw data points (a slight vertical jitter was added for clarity) and coloured according to thermal plasticity. The black bars above each point cloud indicate the mean value of plasticity for plants having and not having any fitness and for each quartile of the distribution of April temperatures.

Among-year variation in single events of selection on flowering time plasticity was correlated with spring temperatures. Less plastic individuals had a larger fitness advantage in colder springs (the effect of the interaction April temperature × individual plasticity of flowering time on yearly individual fitness was credibly different from zero in a generalized linear mixed model including also plant size; figure 3 and table 3). The credible intervals for the effect of the interaction April temperature × BLUP slope estimate did not overlap zero for the zero-inflated part of the models, but overlapped zero for the count part, indicating that effects of temperature on the covariation between reaction norm slopes and fitness acted via the probability of producing any seeds, rather than via the number of seeds produced by individuals producing seeds (table 3). When not adjusting slope estimates for differences in average flowering time, the pattern was the opposite, and more plastic individuals had a larger fitness advantage in colder springs, with effects of temperature acting also only via the probability of producing any seeds (electronic supplementary material, table S3).

**Table 3.** Results of a generalized linear mixed model (zero-inflated negative binomial distribution) with fitness for each individual and year as the response variable, and April temperature, the residuals of the BLUP slope estimate on the BLUP intercept estimate (as a measure of plasticity independent of average flowering time) and their interaction as fixed effects. Plant vegetative size (mean-centred, square-root transformed above-ground volume) is also included as a fixed effect, and individual is included as a random intercept (number of levels: 791). Effects beginning with ‘zi’ represent the zero-inflation part of the model, while other effects represent the negative binomial part (i.e. count) part of the model. Bayesian *R*^2^ was 0.127.

	posterior mean	posterior s.d.	95% CI	
			lower	upper
group-level effects: individual				
s.d. (intercept)	0.251	0.045	0.159	0.333
s.d. (zi_intercept)	0.176	0.098	0.009	0.366
population-level effects				
intercept	2.139	0.033	2.072	2.201
zi_Intercept	1.010	0.039	0.932	1.089
BLUP slope (residuals)	0.401	0.249	−0.078	0.897
April temperature	−0.006	0.027	−0.059	0.048
vegetative size	0.018	0.002	0.014	0.022
BLUP slope (residuals): April temperature	−0.166	0.203	−0.561	0.225
zi_BLUP slope (residuals)	−1.197	0.335	−1.852	−0.549
zi_April temperature	0.083	0.029	0.027	0.139
zi_Vegetative size	−0.042	0.003	−0.049	−0.037
zi_BLUP slope (residuals): April temperature	0.804	0.249	0.318	1.297

Analyses including individuals flowering in at least 2, 3 or 4 years (electronic supplementary material, tables S4–S12) yielded results similar to those including all individuals. The only difference was that although all models suggested selection for a lower responsiveness of flowering time to temperature, the highest posterior density intervals for selection gradients for reaction norm slopes overlapped zero in models where individuals with one or a few observations were excluded (electronic supplementary material, tables S7–S9 and figure S17).

## 4. Discussion

Our 22-year study in a natural population of the perennial forest herb *Lathyrus vernus* showed significant differences among individuals in both mean flowering time and plasticity of flowering time to spring temperature. Direct selection favoured individuals with lower flowering time plasticity, and total and direct selection favoured individuals that flowered earlier. Among-year variation in selection on plasticity was correlated with climatic conditions, the fitness advantage, in terms of a higher probability of producing seeds, of less plastic individuals being greater in colder springs. Our study suggests both that there is phenotypic selection on thermal plasticity of flowering time, and that temperature conditions during spring influence selection on plasticity. Assessing natural selection on plasticity and identifying its drivers using data from natural populations are fundamentally important to understand and predict how organisms adapt to changing environments.

Identifying correlations between mean phenology and plasticity of phenology is essential because their presence implies that selection cannot act independently on each of them. In our study, both average flowering time and flowering time plasticity to average spring temperature differed among individuals. Flowering time plasticity to the frequency of very cold days also differed among individuals, indicating that the effect of average spring temperature might be related to low temperatures and freezing conditions having strong detrimental effects on development, while variance in spring temperature *per se* seemed to be less important (electronic supplementary material, figure S17). The among-individual variance in plasticity to temperature found for flowering time in *L. vernus* is slightly lower than that of another phenological study for laying date in collared flycatchers [22]. It is difficult to say if this difference represents more general differences among organism groups due to the scarcity of studies that have quantified among-individual variation in thermal reaction norms in natural populations [19] (but see [54] for variation among family lines in plants grown under controlled conditions, and [55] for variation across environments using a genetic reference panel in a model species). We also found a strong positive correlation between reaction norm intercepts and slopes, meaning that individuals that on average flowered early were more plastic than individuals flowering late. This pattern is opposite to the suggestion that individuals that on average flower late should be more plastic because a later mean flowering allows for more variation around that mean [56]. The pattern we found for *L. vernus* is, however, similar to the one found for the common gull, where early laying birds were more plastic than late-laying [57]. This shows that the relationship between phenotypic mean trait values and magnitudes of trait plasticity can differ in direction.

We found significant direct selection on both reaction norm intercepts and slopes. Selection gradients showed that plants with an earlier average flowering start and plants with lower responsiveness to spring temperature had higher fitness. Our finding of direct selection against plasticity suggests that the costs of a high plasticity in terms of expressing suboptimal phenotypes are comparatively large in current environments [12]. A potential explanation for why the optimal plasticity is lower than the current plasticity in our study system is that recent increases in spring temperature have shifted the optimal flowering time to be on average earlier across all temperatures, and the optimal plastic response to higher temperatures during early spring to be weaker. For our study site, it is also possible that a successional change from a wooded meadow to a closed deciduous forest over the last century [58,59] has shifted both optimal mean flowering time and optimal flowering time plasticity. When the canopy is fully developed, light availability decreases dramatically compared to the levels before leaf out (J. Ehrlén, unpublished data). For *L. vernus*, which flowers shortly before canopy closure, this means that the period when net assimilation is possible is rather short. In early emerging spring plants, phenology has been found to rely more on temperature than on photoperiod [60], and tree leaf-out is strongly dependent on photoperiod [61]. Thus, if canopy trees rely more on photoperiod as a cue for development than *L. vernus*, then this implies that earlier mean flowering and lower plasticity to temperature should be more favoured in forest understoreys than in more open habitats. Direct selection on reaction norm slopes was not credibly different from zero in analyses where individuals with one or a few observations were excluded, albeit estimates were also positive in these models. Our results are thus consistent with the findings of a previous simulation study, showing that exclusion of individuals with one or a few observations reduces power to detect variance in plasticity and increases the confidence intervals around variance component estimates [41]. Based on the findings of that study, ‘individuals with one or a small number of repeated measures should not be dropped from random regressions' [41]. A plausible interpretation of the results of the different models is thus that censoring individuals flowering in only one or a few years reduced the power of the analyses.

Evidence for phenotypic selection on plasticity from other species is still sparse [19]. However, our finding that selection favoured lower thermal plasticity agrees with some recent animal studies [62,63], although several other studies have found selection only on the intercept, but not the slope of reaction norms of timing of reproduction [64,65]. Importantly, many previous assessments of selection on thermal plasticity might be inaccurate because the analyses did not distinguish between direct selection on plasticity and indirect selection acting through mean phenology [20,22,25,26,28]. In our study, the strong correlation between early flowering and high plasticity of flowering time, in combination with selection for early flowering, led to negative indirect selection on plasticity. As a result, there was no total selection on plasticity. Our study is one of the first to use multivariate RRMMs of traits and fitness to estimate direct and indirect selection on plasticity [19,24,44]. This is an important step, as separating direct and indirect selection on plasticity is necessary to understand selection on both means and plasticity of traits [12]. For example, it has been suggested that the often-observed selection for early flowering might be a result of selection acting on plasticity and plasticity being correlated with mean flowering time [56]. This is not true for our study species, as we found direct selection for an earlier average flowering (i.e. for a lower intercept of the reaction norm) in analyses accounting also for differences in plasticity among individuals. Our assessment of selection on plasticity also accounted for the fact that large plants in a good condition might be able to both flower early and produce many seeds, by including plant size [66]. Still, we cannot dismiss the possibility that indirect selection mediated by other non-measured traits, such as timing of peak flowering [67] or duration of flowering [68], might have contributed to the observed patterns.

A critical question in the face of climate change is whether evolved levels of plasticity will be sufficient to cope with the altered conditions. A recent review showed that optima for timing of reproduction in birds and mammals typically fluctuate, but that phenotypic plasticity enables individuals to largely track these fluctuating optima, thereby reducing phenotypic selection [69]. Another review found that although thermal plasticity indeed has important effects on phenotypic responses, it seems insufficient to keep up with current rates of warming [70]. On the other hand, several recent studies have shown that current levels of thermal plasticity might be too strong, overshooting the optimum phenotype [71,72]. If plastic responses are too strong, then we expect selection to favour reduced thermal plasticity or canalization and phenotypic robustness. Evidence of fitness benefits of lower plasticity has been found not only in the current study, but also in studies with insects and lizards [62,63]. Taken together, the available evidence thus suggests that plasticity should not *a priori* be assumed to be adaptive, and that increased temperatures can lead to selection for stronger, weaker, as well as maintained levels of plasticity.

Identifying the agents influencing selection on phenotypic plasticity is necessary to understand and predict responses to environmental changes. A few previous studies have examined how environmental factors influence selection on plasticity of timing of reproduction in plants [26,29,30]. However, our study is the first to link variation in selection on thermal plasticity of flowering time in natural populations to among-year differences in climatic conditions. We found that selection against high responsiveness to spring temperature was stronger in cold springs, and that less plastic individuals more often produced seeds than more plastic individuals in these years. Our results thus suggest that spring temperature is an important selective agent on plasticity of flowering time. One possible explanation for the observed increased selection against plasticity in colder springs is that given a similar mean flowering time, less plastic flower earlier in cold springs, and that this is beneficial because it allows capitalizing on favourable light conditions before canopy closure while the risk of being exposed to unfavourable climatic conditions is relatively small in cold springs with late mean development. It is also possible that the selection for lower flowering time plasticity might be driven by differences in timing relative to canopy closure. If canopy trees rely on photoperiodic cues to a larger extent than *L. vernus* [60,61], then trees would be less delayed than *L. vernus* in years with lower than mean spring temperatures. This in turn would favour a less plastic response in *L. vernus* in order to ensure sufficient time for assimilation and seed provisioning before the canopy closes. Lastly, the fact that variation in flowering time among individuals differing in plasticity is relatively small in cold springs (figure 1) suggests that the relative advantage of a low plasticity in cold springs might also be due to plasticity carrying costs that are expressed under all conditions, whereas the benefits are only present in warm springs when highly plastic individuals further advance their flowering time. Notably, when not adjusting for differences in average flowering time, more plastic individuals were instead favoured in cold springs (electronic supplementary material, table S3). This was because more plastic individuals on average flowered earlier, and early flowering individuals were favoured in cold springs.

Our study was observational, and without experimental manipulations it is not possible to conclusively identify the causal link between April temperature and selection on plasticity. In our study system, it is possible that spring temperature influenced selection both directly, and indirectly through effects on biotic interactions, such as grazing and seed predation [35]. Spring temperature influenced the relationship between plasticity and the probability of producing any seeds, but not the relationship with the number of intact seeds. In our study system, grazing can be one cause of such complete reproductive failure. In our analyses, we included also individuals that were grazed before flowering and estimated their expected first flowering date in order to account for the fact that some individuals (the invisible fraction [39]) were exposed to factors influencing fitness before expressing the trait of interest. It is therefore possible that grazers mediated some of the effects of spring temperature on selection by preferentially attacking plants with a certain phenology [35]. Our findings of selection against high plasticity in flowering time being strongest at low temperatures agree with the results for other species showing that more responsive genotypes have higher fitness under elevated temperatures [26]. However, selection against plasticity in flowering time at high temperatures, possibly as a result of that temperature stress influences the relationship between plasticity and fitness, has also been found [25]. In our study system, stress due to high temperature or drought is not likely to be important as April temperatures are mild and availability of water is high.

## 5. Conclusion

Studies using field data from natural populations are indispensable to assess phenotypic selection, as selective agents and selection might differ considerably from natural conditions in studies performed in greenhouses or common gardens. Our long-term field study showed that there is phenotypic selection on individual thermal plasticity of flowering time, and that this selection depends on climatic conditions in terms of spring temperatures. This suggests that climatic changes are likely to change not only phenotypic and genotypic mean values of life-history traits, but also their plasticity, i.e. the way in which the timing of life-history events responds to short-term climatic variation. Identifying agents of selection on plasticity is thus fundamentally important to understand the role of phenotypic plasticity in evolutionary responses to environmental changes, and to predict long-term responses to ongoing changes in climate.

## Data Availability

The data from this study are available from the Dryad Digital Repository: https://doi.org/10.5061/dryad.j6q573njx [73]. Supplementary material is available online [74].
